# “You could lose when you misuse” – factors affecting over-the-counter sale of antibiotics in community pharmacies in Saudi Arabia: a qualitative study

**DOI:** 10.1186/s12913-018-3753-y

**Published:** 2018-12-03

**Authors:** Faten Alhomoud, Reem Almahasnah, Farah Kais Alhomoud

**Affiliations:** Department of Pharmacy Practice, College of Clinical Pharmacy, Imam Abdulrahman Bin Faisal University, Dammam, Saudi Arabia

**Keywords:** Non-prescription, Self-prescription, Self-medication, Over-the-counter, Misuse, Overuse, Antibacterial, Antibiotic, Saudi Arabia

## Abstract

**Background:**

The sale of antibiotics without a prescription poses a global public health concern. Antibiotics dispensed without a prescription are largely recognised as a cause of antibiotic misuse and overuse which may result in antibiotic resistance, recurrent infection, increased cost and adverse effects of treatment. There have been no qualitative studies to explore the reasons for over-the-counter (OTC) sale of antibiotics, despite the fact that non-prescription sale of antibiotics are increasing in Saudi Arabia.

**Methods:**

Qualitative interviews were conducted with community pharmacists living in the Eastern Province of Saudi Arabia using face-to-face, open-ended questions. Interviews were audio-recorded and transcribed verbatim. The interview transcripts were analysed using thematic analysis and NVivo 10 software.

**Results:**

All participants declared that antibiotics were frequently sold without a medical prescription on an OTC basis. The main reasons for OTC sale of antibiotics were found to be related to the ease of access to community pharmacies compared to other healthcare services, expertise and knowledge of pharmacists and patients’ trust, misconceptions and inappropriate practices of the public towards antibiotic use, customer pressure, pharmacists’ need to ensure business survival and weak regulatory enforcement mechanism. These are presented in more detail below by using illustrative quotes from participants’ transcripts.

**Conclusions:**

The non-prescribed sale of antibiotics is still a common practice in Saudi Arabia, despite being a problem. The results of this study highlight the need to design interventions to promote rational use of antibiotics.

**Electronic supplementary material:**

The online version of this article (10.1186/s12913-018-3753-y) contains supplementary material, which is available to authorized users.

## Background

Even though antibiotics represent a vital public health achievement, their overuse and misuse, as a result of irrational prescribing and dispensing and self-medication, may cause serious adverse events, recurrent infections, antibiotic resistance, and increased treatment cost and poor health outcomes [1–5]. It was estimated that 23,000 deaths and more than 2 million illnesses were caused by antibiotic resistance [5]. A review estimated that, by 2050, antimicrobial drug resistance (AMR) would lead to 10 million people dying every year unless the problem of AMR is tackled by a global response [6].

In Saudi Arabia, the availability of antibiotics over-the-counter, without medical prescriptions, is a major contributing factor towards self-medication behaviour among the general population [7]. Self-medication includes the use of medicines by the consumer to treat self-recognised symptoms or disorders without proper medical consultation [8]. Studies in Saudi Arabia reported that non-prescribed sale of antibiotics was high in community pharmacies and ranged from 63 to 82% [9–12]. Although dispensing antibiotics without a prescription has been illegal for pharmacists for more than three decades in Saudi Arabia [13], studies revealed that over half of the pharmacists dispensed antibiotics without prescription to treat cold, cough, sore throat and other viral infections [3, 9–12]. Others dispensed antibiotics without a clear clinical indication and without carrying out any patient counselling [3, 9–12].

In Saudi Arabia, the rates of both antibiotic consumption and resistance are amongst the highest in the Middle Eastern countries [3, 14, 15]. Thus, self-medication – and irrational antibiotic dispensing and prescribing – is causing a major concern to the public health sector and Ministry of Health (MoH) in Saudi Arabia [3, 14, 15]. A systematic review conducted in the Middle East, by Alhomoud et al., reported that self-medication with antibiotics was high in Saudi Arabia and ranged from 48 to 79% [3]. Based on another review conducted from 1990 until 2011 in Saudi Arabia, the prevalence of gram-negative resistant bacteria ranges from 7.6 to 92.3%, with *Klebsiella pneumoniae, Acinetobacter species* and *Pseudomonas aeruginosa* showing the highest prevalence, whereas the prevalence of gram-positive resistance bacteria ranges from 23.5 to 30.7%, with *Clostridium difficile*, *Methicillin-resistant Staphylococcus aureus (MRSA)* and *Streptococcus pneumoniae* having the highest prevalence [14].

Pharmacists often serve as the first contact for the patient in the healthcare-seeking chain and the last before consumption of the drug dispensed [16]. They are also easily accessible healthcare providers and are ideally positioned to convey health promotion messages [16]. Most of the previous studies in Saudi Arabia focused on community pharmacists’ dispensing practices and their role in facilitating self-medication and the potential risks associated with this practice [9–12], but studies on community pharmacists’ perceptions towards reasons behind OTC sale of antibiotics using an in-depth exploration are lacking [17]. Thus, it is important to explore reasons for non-prescribed sale of antibiotics from the pharmacists’ perspectives in Saudi Arabia because their dispensing practices may facilitate self-medication and increase the risk of antibiotic resistance. The findings from this study would help healthcare professionals to develop strategies to encourage safe and rational use of antibiotics in the community pharmacies.

## Methods

### Study setting, recruitment and data collection

A qualitative exploratory study design, which used face-to-face interviews with community pharmacists in the Eastern Province of Saudi Arabia, for the period from January to May 2017, was conducted.

The study population consisted of registered community pharmacists living in the Eastern Province of Saudi Arabia, and who were able to speak Arabic or English. A convenience sampling technique based on locating pharmacies near to the researcher’s area of residence was used to select the pharmacies. Once identified, a personal visit was made to each community pharmacy to inform staff about the study and invite the in-charge pharmacist to take part. If the in-charge pharmacist agreed to participate, a face-to-face in-depth interview was conducted on the spot in the pharmacy. Data collection was continued until the desired sample size was achieved. The sample size was determined by the principle of saturation. Interviews took place until no new themes emerged. The saturation point was met after completing 20 face-to-face interviews.

An open-ended interview guide with probing on existing dispensing practices was used (see Additional file 1: Appendix 1). The interview guide was developed after undertaking a literature review of research conducted in the Middle East to identify reasons for OTC sale of antibiotics. The interview guide was divided into two main parts. The first part focused on pharmacists’ characteristics (e.g. age, gender, nationality, educational level, pharmacy professional’s age, etc.). The second part was designed to explore the professionals’ views on the reasons behind OTC sale of antibiotics and current antibiotics dispensing practices. One pharmacist with previous experience in qualitative data collection techniques was trained to conduct the interview. Verbal and written documentation of informed consent was obtained from all participants before beginning the interviews. The interviews were all conducted in Arabic, based on the participants’ preference. The average duration of the interview was 30 min, ranging from 15 to 45 min. Interviews took longer when customers interrupted them. Interviews were audio-recorded, transcribed verbatim and analysed thematically using NVivo 10 software.

### Data management, analysis, validity and reliability of analysis

The audio-recorded interviews were translated from Arabic to English. Five of the recordings were transcribed and translated by a bi-lingual pharmacist and compared with the primary work to check the accuracy of the translation. Interview transcription and analysis occurred concurrently with the data collection process to enable a judgement to be reached regarding data saturation. Data saturation was reached when new information no longer arose and the study provided the maximum information on the phenomenon. The interviews were analysed thematically using an inductive approach. The thematic analysis was used to assess the interviews and classify data according to key categories and themes. The inductive approach was taken to allow research findings to emerge from the dominant, frequent or significant themes by grouping descriptions from open questions. In the analysis, data are sifted, charted and sorted in accordance with key themes and issues in a systematic process. This was conducted in order to facilitate transparent, rigorous and systemic management of data, allowing the researchers to move back and forth between different levels of abstraction without losing sight of the ‘raw’ data. To facilitate coding, and to search and retrieve data, a computer assisted qualitative data analysis software (CAQDAS) (NVivo10) was used. The software allowed searching and retrieval of particular segments of texts for inspection, storing the transcribed text in an organised form, coding of data, linking data to form themes or categories, writing memos, and drawing conclusions and verification.

A primary coding structure was developed by the research team by using a subset of data which reflected participants’ descriptions of issues and reasons behind OTC sale of antibiotics. To ensure a reliable, robust and accurate coding procedure, these primary codes were derived independently by one researcher external to the study and two members of the research team. In case of deviations, these were discussed and the coding frame refined to ensure a consistent procedure.

## Results

Twenty pharmacists (of a total of 29 invited to do so) participated in the current study. The response rate for completed interviews was 69%. A description of the participants’ characteristics is shown in Table 1.

**Table 1:** Characteristics of participants recruited into the study

Parameter		Results
Age (years)	Mean (SD)	33 (5.86)
Years of work experience	Mean (SD)	10 (3.22)
Gender [N (%)]	Male	20 (100)
Nationality [N (%)]	Non-Saudi	20 (100)
Education [N (%)]	Bachelor	19 (95)
	Master	1 (5)

All pharmacists reported dispensing antibiotics without prescription mainly for the following indications: fever, sore throat, cold/flu and cough. The most commonly dispensed antibiotics were Amoxicillin-Clavulanic Acid (Augmentin), Amoxicillin and Azithromycin. Interviews revealed that various reasons contribute to the rise of OTC sale of antibiotics, ranging from the ease of access to community pharmacies compared to other healthcare services, expertise and knowledge of pharmacists and patients’ trust, misconceptions and inappropriate practices of the public towards antibiotic use, customer pressure, pharmacists’ need to ensure business survival and weak regulatory enforcement mechanism. These are presented in more detail below by using illustrative quotes from participants’ transcripts.

### Factors contributing to OTC sale of antibiotics from pharmacists’ perspectives

#### Community pharmacies’ accessibility

##### Time and financial cost

Some pharmacists reported that patients prefer to visit community pharmacies rather than hospitals to obtain antibiotics due to their easy access. According to those pharmacists, visiting community pharmacies not only save patients’ time by avoiding the long queues associated with waiting for a consultation or for their prescriptions to be filled or dispensed, it also saves the money that would be spent on a consultation, especially in private hospitals.

“If you go to a private hospital, they’ll charge you for both the visit and the money for the medicine, so that’s more money.” [P11]

“… in hospitals, patients have to wait longer to see a physician, while in a pharmacy patients will explain their condition and then immediately will get the antibiotic.” [PH19]

One pharmacist revealed that, even if a patient has health insurance and thus he/she does not have to worry about the cost of the consultation or the medicine, these costs are still off-putting. Visiting a private hospital was thought of as troublesome by patients even if they were insured, because of the long waiting times, as it may take three to four hours to see a doctor, or in terms of the longer distances that have to be travelled:

“Some patients want to save time, even if they have an insurance card, they will not go to hospitals as the waiting time is three to four hours. It is also convenient here in the pharmacy. Pharmacies are close to everyone whereas, if you go to hospitals, you would have to drive for a while. Here [pharmacy] there are no registration and no waiting in line required.” [PH7]

##### Iqama (residency permit) or health insurance problems

To provide appropriate healthcare services for expatriate workers in the private sector in Saudi Arabia, the Compulsory Employment-Based Health Insurance (CEBHI) was introduced. The CEBHI was implemented in order to improve utilisation of the government healthcare budget and ensure and regulate the provision of healthcare for expatriates (while providing financial protection against their healthcare expenses), by increasing the contribution of private healthcare sector expenditure, and reducing the load on government healthcare professionals [18]. Although, according to the Saudi Labour Law [18], employers must cover expatriate employees by paying all necessary medical expenses for them, two pharmacists mentioned that some expatriate workers are unable to access private hospitals due to the lack of insurance or the inability to obtain or renew their Iqama (residency permit). Thus, such patients find it much easier to approach a pharmacy and ask for an antibiotic they require without the need to provide either a valid Iqama or health insurance. One pharmacist said:

“Many expatriate workers have the problem of not having a valid Iqama or private insurance. This means that they can’t go to either a private or even government hospitals. That’s why many of them come straightaway to pharmacies.” [PH3]

##### Time available in the consultation

Lack of time given to patients to discuss their worries and concerns with their doctor was frequently seen by pharmacists as a major limitation on the quality of the consultation. Some pharmacists perceived the consultation time patients spend with their doctor as ‘rushed’ due to the perception of the doctor’s time as ‘precious’ and the doctor being a ‘busy person’, which puts pressure on the patients, leaving them unable to express all their health concerns and worries. Patients feel that doctors are not willing to listen or pay attention to their complaints. Pharmacists also believed that they have more time to spend and can direct interaction with the patient to counsel them appropriately regarding their complaints and medications, unlike physicians. These thoughts are illustrated in the following comments:

“Physicians are busy as they meet new patient every 15 minutes, while the pharmacy is open and patients can come at any time to explain their problems and get immediate solutions.” [PH1]

“There are patients who meet physicians and get medicine, then throw it in the garbage as the physicians couldn’t even be bothered to listen to them. Patients feel like they are taking up his/her time… that makes them feel under pressure. That’s the reason why they prefer to come to pharmacists.” [PH19]

### Expertise and knowledge of pharmacists and patients’ trust

Some pharmacist said that they would provide antibiotics without a medical prescription by applying their professional judgement. They felt that they were qualified, experienced, well trained and knowledgeable to diagnose and treat various infections:

“There are many cases where it is safe to prescribe antibiotics. I worked in the hospital for 12 years and I have the knowledge and capabilities to prescribe. Rather than the Ministry of Health prohibiting pharmacists dispensing antibiotics without a prescription, they should activate the role of the pharmacists as they are part of the medical team.” [PH1]

One pharmacist suggested that the Ministry of Health’s current laws should be modified in a way that enabled the pharmacists to prescribe certain antibiotics in mild to moderate cases after assessing the patient’s condition according to their experience and knowledge:

“The current laws of the Ministry of Health should be modified in which a physician has to diagnose the condition and send the diagnosis to me in order to prescribe the suitable antibiotic and dispense it to the patient, as I didn’t study all these years in order to read the prescription and give the patient the prescribed medicine.” [PH12]

One pharmacist reported that there are areas of professional activity where, by good practice and law, the pharmacist can practise independently without the need to consult a doctor, such as in dispensing antibiotics. This pharmacist revealed that even physicians who are experienced and licensed to prescribe antibiotics may prescribe them inappropriately due to lack of testing or irrational prescribing patterns:

“When a patient comes to me, I believe that I am qualified and well trained to dispense antibiotics to him/her without consulting a doctor. I know what I’m saying is against the government policy but we are not living in an ideal world. In many situations, some physicians prescribe antibiotics without any proper diagnosis such as ordering lab tests or cultures, which is against their profession. If the government wants to control irrational use of antibiotics, they should control physicians’ irrational prescribing first.” [PH8]

Most pharmacists who felt that they had the knowledge and experience declared that they would only feel confident to manage certain cases of infections, such as throat infection, wound infection, or urinary tract infection, whereas complicated infections require a consultation with a physician.

As reported by some participants, community pharmacists are the healthcare professionals that are most accessible to the public. They also provide valuable information and free counselling to patients at the time they dispense the prescription and non-prescription drugs. They maintain links and relationships with their patients in the community which lead to trust developing between them in primary care. According to one pharmacist, patients are worried when a physician prescribes a certain antibiotic over another. This is based on the fact that when prescribing antibiotics some physicians are guided by the name of a pharmaceutical company or specific brands and not the evidence-based practice. This is because pharmaceutical companies’ representatives spend all day in the hospitals, promoting their commodities and their brands in any way possible. They may also give a commission to physicians to market their brands, which induces physicians to write unnecessary prescriptions or go for one brand over another without medical justification, and this results in patients losing trust in physicians.

“Patients trust pharmacists more than physicians for the following reasons: firstly, pharmacists provide free consultations, thus pharmacists are considered to be the cheapest consultants. Secondly, many patients are concerned that drug marketing to physicians might influence their prescribing practices. Physicians have become businessmen, their prescribing decisions and choices of drugs are influenced by drug promotion by medical representatives. Pharmaceutical companies have representatives in every hospital to promote their medications. They do sales visits and events that may involve gifts and commissions to physicians to sell their brands. Due to this, patients have turned to the community pharmacists which they perceive to provide them with free care and appropriate medication dispensing and counselling without being influenced by pharmaceutical companies’ promotions.” [PH7]

Another pharmacist revealed that some physicians tend to over-prescribe antibiotics which may not be necessary for certain patients. This issue makes patients feel doubtful about doctors’ decisions and thus prefer to consult pharmacists.

“Some physicians prescribe antibiotics more than needed and patients may or may not need it [antibiotic]; this is one of the reasons why patients lose trust in physicians and prefer to consult pharmacists.” [PH8]

### Misconceptions and inappropriate practices of the public in respect of antibiotic use

Some pharmacists reported that the Saudi Arabian public had misconceptions about and conducted inappropriate practices with regards to antibiotic use. Some members of the public believed that antibiotics can eradicate and treat any infection and speed-up recovery. Having a strong belief in the curative power of antibiotics had led some of the general population to use antibiotics when not indicated, to treat viral and other self-limiting illnesses:

“There are some patients who are convinced that if they don’t take [an] antibiotic, their illness will never subside; they insist on taking the antibiotic even if they don’t need it. To them, it is similar to Panadol. It is not about that I don’t want to dispense the antibiotic, but they have to let their immune system fight against the infection naturally.” [PH14]

As reported by one pharmacist, antibiotics were also used by patients because they believed that they have the ability to ‘prevent’ infections:

“Many patients prefer to come to the pharmacy for antibiotics in order to avoid infections.” [PH16]

Pharmacists also reported that, when some patients experience recognisable symptoms that they have developed before, they request the treatment that successfully treated their symptoms during previous episodes. One pharmacist reported that patients had usually sought a diagnosis from a healthcare provider, often a doctor, for the first episode of a specific illness. If patients had a subsequent episode with a similar symptom, they felt comfortable with a self-diagnosis. Thus, self-medication seems suitable because they are depending on prior experience with similar symptoms to identify the condition, make a diagnosis and decide upon treatment, a behaviour which may have substantial public health implications:

“A patient will not request a specific antibiotic unless he used it in a recurrent infection; it means previously a physician had prescribed the antibiotic and it cured the infection. For example, he got tonsillitis and went to the doctor one time and the doctor prescribed Augmentin for him, which relieved his symptoms in a few hours. Thus, whenever he gets the same infection, he will buy the same antibiotic from the pharmacy as no patient is willing to pay 100 SR unless he has already tried it.” [PH7]

Pharmacists also revealed that the public had a lack of awareness relating to development of antibiotic resistance, antibiotic allergies and adverse effects of antibiotics.

### Customer pressure and pharmacists’ need to ensure business survival

Some pharmacist had felt under pressure from customers to dispense antibiotics. Pharmacists reported receiving requests from patients to dispense an antibiotic even though there was no medical indication for its use. Some pharmacists did not always fulfil these requests. However, others sometimes did dispense an antibiotic in such cases, mainly because of patient pressure or because they were worried that they would lose a customer, as this patient may go to another pharmacy for the antibiotics, which may affect the first pharmacist’s business:

“The pharmacy owner is not pushing the pharmacist to dispense antibiotics, it is all coming from patient pressure. When I refuse to give them [customers] an antibiotic, many customers say that they will get it from another pharmacy. That’s why I still dispense without a prescription.” [PH9]

“Many patients insist on getting antibiotics although they don’t need them, and I do not want to lose a customer. You can see many pharmacies around here sell antibiotics without prescriptions.” [PH3]

Some pharmacists reflected that OTC sale of antibiotics is wrong but they had to do it otherwise their business would be affected, as nearby pharmacies would sell antibiotics without a prescription. One pharmacist highlighted this:

“If a patient comes with an empty box and I know him/her well, then I dispense it for him/her. I know it is illegal and wrong to do, but if I stop dispensing, it will affect the business.” [PH6]

### Weak regulatory enforcement mechanism

Some pharmacists indicated that people will buy antibiotics directly from pharmacies without visiting a doctor; this is because the enforcement of regulations is weak and antibiotics are often available and easily accessible in the pharmacies and are sold without a medical prescription. Pharmacists indicated that the selling of antibiotics without a medical prescription is not because of a lack of awareness or knowledge about the consequences of irrational antibiotic use but due to the lack of a strong regulatory enforcement mechanism. One pharmacist called for strong regulatory mechanisms to control OTC sale of antibiotics, such as penalising violators:

“There is a weak regulation system. That’s why there is a continued and expanding sale of OTC antibiotics. If the Ministry of Health put in place a regulatory mechanism and enforced penalties, this would help in reducing the OTC sale of antibiotics. If I know that my licence could be revoked and the pharmacy could be closed, I would never sell antibiotics without prescription.” [PH15]

Although some pharmacists thought that strong regulatory mechanisms and law enforcement would help in controlling OTC sale of antibiotics, other pharmacists felt that educating the public about the consequences of antibiotic misuse and resistance was more important than enforcing the laws. This can be done by sending messages and conducting awareness campaigns which may help patients in making informed choices and decisions about buying or using antibiotics:

“Raising patient awareness via mobile messages or campaigns in malls or schools is very important as the patient is the one who will take the final decision. Thus, increasing the awareness of the patients is more important than enforcing the laws. Whatever rules you put for pharmacists, the patient is the one who will make the final decision. Thus, they need to be informed.” [PH9]

## Discussion

This article presented the first qualitative study exploring a broader range of factors influencing OTC sale of antibiotics and it confirms that the sale of antibiotics without prescription is a common practice in Saudi Arabia. These results are in line with other studies conducted in Saudi Arabia, which reported that non-prescription sale of antibiotics is common and almost all pharmacists were willing to sell antibiotics without a prescription [9–13]. This practice has also been observed in different Middle Eastern countries such as Iran, Turkey, the United Arab Emirates, Kuwait, Oman, Yemen, Jordan, Syria, Lebanon [3] and Egypt [19]. Inappropriate use of antibiotics can put patients at risk of antibiotic resistance, increased healthcare costs and mortality rate, poor health outcomes, and adverse drug reactions and contraindications [20, 21].

In the current study, pharmacists disclosed that they sold more Amoxicillin-Clavulanic Acid (Augmentin), Amoxicillin and Azithromycin without prescription, which was consistent with other studies conducted in Saudi Arabia [9–13, 17]. However, Ciprofloxacin was also found by these studies to be one of the most commonly dispensed antibiotics without prescription [9, 17]. This variation can be due to variations in the clinical scenarios presented to the pharmacists. Sore throat, common cold/flu and cough and fever accounted for the highest rate of antibiotic sale without prescription, which was in line with other studies conducted in Saudi Arabia [3, 9–13]. Using antibiotics for inappropriate conditions represents an issue in Saudi Arabia [3, 9–12]. The leading condition for which antibiotics are misused is upper respiratory tract infections, most of which are usually viral [3, 9–12]. This was consistent with the current study findings where the leading indications for which antibiotics were dispensed were for upper respiratory symptoms that are mostly viral and self-limiting. This can lead to increased consultations, adverse effects, high cost and increased risk of resistance [1–5].

An exploration of factors influencing OTC sale of antibiotics among study participants revealed similarities with evidence from the previous Saudi studies [3], although some differences were also found. For instance, lack of a strong regulatory enforcement mechanism to control OTC sale of antibiotics [3, 9–12], expertise and knowledge of pharmacists [11], time and financial cost saving [3] tied well with Saudi literature and were all found to be possible reasons for non-prescription sale of antibiotics. The possible causes of non-prescription sale of antibiotics that vary from Saudi literature were patients’ trust, Iqama or health insurance problems, time available for consultation, misconceptions and inappropriate practices of the public in relation to antibiotic use, customer pressure and pharmacists’ need to ensure business survival. A study from Ethiopia indicated that the customer pressure, professional conflicts of interest, owner’s interest in maximising revenue, together with improper enforcement of drug regulations were all important reasons for increasing non-prescription sale of antibiotics [22]. Another study, conducted by Black et al. in Qatar, also mentioned that professionals’ and the public’s lack of knowledge and awareness about rational antibiotic use as well as the professionals’ desire to meet customer demand were reported as the major reasons for over-the-counter sale of antibiotics [23].

Some pharmacists requested strict law enforcement and community awareness campaigns to control or limit non-prescription sale of antibiotics and raise public awareness regarding the consequences of antibiotic misuse, which may help change people’s cultural belief and practices in relation to the widespread behaviour of self-medication. Therefore, strong regulatory enforcement to prohibit the sale of antibiotics over-the-counter at pharmacies should be promoted. It is necessary to develop surveillance on sale of antibiotics in community pharmacies and establish a penalty system for the illegal antibiotics sale. Strict regulatory enforcement has greatly reduced the dispensing of illegal antibiotics in other countries. For example, after the effective enforcement of a regulation on antibiotic sale, the OTC antibiotic dispensing rate was lowered in private pharmacies in Zimbabwe [24]. Strengthened regulations, law enforcement and organising awareness-creating campaigns with a special focus on the public were also recommended by studies conducted in South Korea and Chile [25, 26]. In these studies, the regulatory enforcement measures and simultaneous public education campaign have been shown to have a greater effect on limiting OTC sale of antibiotics and have improved the antibiotic resistance profile [25, 26].

Although some pharmacists highlighted the need for strict enforcement of laws, the findings also drew attention to the ease of access to community pharmacies compared to other healthcare services, which indicates the important role that pharmacists can play in informing and educating patients about the appropriate antibiotics use. Pharmacists in the current study also suggested a need to expand their role from drug seller to more effective healthcare professional to improve the use of antibiotics. Pharmacists are key health providers with the knowledge, training and skills required to contribute to the reduction of OTC sale of antibiotics and self-medication practices.

Pharmacists in many European countries already have the capacity to take on extra responsibilities and roles to improve the rational use of antibiotics. For instance, in the United Kingdom a new activity is being piloted [27, 28] whereby certain pharmacies are conducting point-of-care testing (PoCT) linked to sore throat and patients are supplied with penicillin-V (or, in case of allergy, clarithromycin) if bacterial tonsillitis infection is detected. This activity permits some registered pharmacists to administer and/or supply specified medicine(s) to a predefined group of patients, without consulting a doctor [16, 29]. Several point-of-care tests have been shown to be effective in lowering the number of antibiotics prescribed [30].

Pharmacists are in a great position to promote the rational use of antibiotics among their customers since patients frequently visit pharmacies to obtain over-the-counter antibiotics. The community pharmacist, for instance, should conduct prudent dispensing, promote appropriate antibiotics dispensing, and increase patient awareness of the importance of avoiding self-medication without appropriate diagnosis and of the alarming problem of antibiotic resistance [27]. With patient counselling, pharmacists are also well placed to detect and correct any wrong concerns and beliefs patients might have, counsel patients on appropriate use of antibiotics and the danger of antibiotic resistance and adverse effects, give patients advice on how to manage symptoms without antibiotics, and apply best infection prevention and control practice. For example, it is vital to educate patients about the natural history of infectious diseases. Patients need to know that, in most self-limiting infections, antibiotics are probably not going to be useful, and that the antibiotic treatment is associated with substantial side effects and risks [31]. They should also be notified that they should only seek care if they have worsening or alarming symptoms such as high fever, shortness of breath, etc. Information brochures are useful to deliver such messages. In order to inform the population on the practices that promote the emergence of antibiotic resistance, media professionals need to get proper training on how to deliver scientific and medical messages in language that is understandable to the lay population through use of multiple channels [32]. Education programmes have been shown to be beneficial in the fight against HIV/AIDS in many countries and a similar approach to antibiotic resistance will likely result in an added benefit [33, 34].

### Strengths and limitations

Strengths: (1) this article presented the first qualitative study exploring a broader range of factors influencing OTC sale of antibiotics. Limitations: (1) the sample of this research consisted of people who were living in the Eastern Province of Saudi Arabia; therefore, careful attention must be paid before transferring the conclusions to people living in other provinces of Saudi Arabia; (2) participants were interviewed in an open area in the pharmacy where the interview could be overheard or interrupted by some distractions presenting in the pharmacy; (3) translating the interviews from Arabic to English may carry an inherent risk of losing the meaning of respondents’ thoughts and perceptions. However, the translation was checked by a pharmacist who had research interviewing experience and health-related qualifications, is bi-lingual (proficient in English and a native speaker of Arabic), and is professionally trained and familiar with the concepts being examined in the study.

## Conclusions

Non-prescription antibiotic dispensing is still a common practice in Saudi Arabia. By uncovering factors influencing OTC sale of antibiotics, the study can provide insight when designing future interventions to promote the safe and rational use of antibiotics. Interventional research studies might be required to assess the effectiveness of the various approaches that could be used to tackle this public health problem.

## Additional file

Additional file 1: **Appendix 1.** Interview guide. (DOCX 61 kb)

## Abbreviations

AMR: Antimicrobial drug resistance
CAQDAS: Computer assisted qualitative data analysis software
CEBHI: Compulsory Employment-Based Health Insurance
MOH: Ministry of Health
MRSA: Methicillin-resistant *Staphylococcus aureus*
OTC: Over-the-counter
PoCT: Point-of-care testing
