# Supplementary material for: “You could lose when you misuse” – factors affecting over-the-counter sale of antibiotics in community pharmacies in Saudi Arabia: a qualitative study
Source: BMC Health Serv Res. 2018 Dec 3;18:915. doi: 10.1186/s12913-018-3753-y (PMC6276151; doi:10.1186/s12913-018-3753-y)
Supplement: Supplementary file 1 — Appendix 1. Interview guide. (DOCX 61 kb) [file 12913_2018_3753_MOESM1_ESM.docx]

**Additional file 1: Appendix 1. Interview guide of pharmacists’ views on the reasons behind OTC sale of antibiotics and current antibiotics dispensing practices**

*Part 1: Pharmacists’ characteristics*

| Item | |
| --- | --- |
| 1. Age |  |
| 2. Gender | - Male |
|  | - Female |
| 3. Nationality | - Saudi |
|  | - Non-Saudi |
| 4. Educational level |  |
| 5. Years of work experience |  |

*Part 2: Exploring pharmacists’ views on the reasons behind OTC sale of antibiotics and current antibiotics dispensing practices*

| Questions |
| --- |
| 1. Do you dispense antibiotics without prescription?  If yes, how often do you do this? When was the last time you did this? |
| 2. What do you think the mostly sold antibiotics without a prescription?  And what is most common indications for antibiotics sold without a prescription? |
| 3. What are the reasons of antibiotics being dispensed without a prescription? |
| 4. Do you consider that the consequences of antibiotic use are conveyed to patients? |
| 5. What are the most important points that you would cover when you counsel a patient about antibiotics sold with/without a prescription? |
| 6. In your opinion, what is the impact of selling antibiotics without a prescription on the health system in Saudi Arabia? |
| 7. What recommendations would you suggest for improving antibiotic use and stopping antibiotic dispensing without a prescription? |
